# Optimization of Aceclofenac Proniosomes by Using Different Carriers, Part 1: Development and Characterization

**DOI:** 10.3390/pharmaceutics11070350

**Published:** 2019-07-18

**Authors:** Rana M.F. Sammour, Muhammad Taher, Bappaditya Chatterjee, Aliasgar Shahiwala, Syed Mahmood

**Affiliations:** 1 Pharmaceutical Technology Department, Kulliyyah of Pharmacy, International Islamic University Malaysia, Kuantan 25200, Pahang, Malaysia; 2 Pharmaceutics Department, Dubai Pharmacy College for Girls, Dubai, UAE; 3 Department of Pharmaceutical Engineering, Faculty of Engineering Technology, University Malaysia Pahang, Kuantan 26600, Pahang, Malaysia; 4 Centre for Excellence for Advanced Research in Fluid flow (CARIFF), University Malaysia Pahang, Kuantan 26600, Pahang, Malaysia

**Keywords:** aceclofenac, proniosomes, niosomes, carrier

## Abstract

In the contemporary medical model world, the proniosomal system has been serving as a new drug delivery system that is considered to significantly enhance the bioavailability of drugs with low water solubility. The application of this system can improve the bioavailability of aceclofenac that is used for the relief of pain and inflammation in osteoarthritis, rheumatoid arthritis, and ankylosing spondylitis. The present study is intended to develop an optimized proniosomal aceclofenac formula by the use of different carriers. Aceclofenac proniosomes have been prepared by slurry method, and different carriers such as maltodextrin, mannitol, and glucose were tried. Prepared proniosomes characterized by differential scanning calorimetry (DSC) analysis and Fourier transform infrared (FTIR) analysis revealed the compatibility of the drug chosen with the ingredient added, powder X-ray diffractometry (XRD) confirmed the amorphous phase of the prepared proniosomes, and finally, the surfactant layer was observed by scanning electron microscopy (SEM). Aceclofenac physical state transformations were confirmed with all formulas but maltodextrin proniosomes exhibited solubility more than other formulations. HPLC method has been used to analyze the niosomes derived from proniosomes in terms of their entrapment capability and drug content. The obtained results revealed that aceclofenac proniosomes can be successfully prepared by using different carriers.

## 1. Introduction

Vesicular systems are becoming leading systems in the field of drug delivery. Scientists are increasingly working on these systems for improving their chemical, physical, and biological properties. Liposomes, proliposomes, niosomes, proniosomes, transfersomes, and pharmacosomes are some examples of the vesicular systems that are attracting much attention due to their flexibility to be tailored for varied desirable purposes [1]. However, due to problems of stability and affordability, liposomes and proliposomes have become unpopular among scholars [2]. According to Kakar et al. [3] niosomes are considered cheaper and easier in terms of preparation if compared to liposomes, however fusion, aggregation, sedimentation, and leakage on storage of such liquid dosage form caused a barrier in front of this attractive vesicle.

The contemporary proniosomes have been designed as a new vesicular system that possesses all the advantages of niosomes and at the same time covering their disadvantages. A study conducted by Hu and Rhodes (1999) [3] reported that proniosomes had advantages over liposomes and niosomes in physical stability characters such as aggregation or fusion of vesicles and leaking of entrapped drugs during long-term storage; also, less chemical degradation problems are encountered with proniosomes. Consequently, it has attracted a lot of attention from researchers, who are focusing on its desirable properties and applications to utilize the best characterizations of the vesicular systems [4].

Proniosomes are dry, free-flowing formulations of a surfactant-coated carrier that is suitable for different routes of administration. Proniosomes are rehydrated by brief agitation in hot water to form a multi-lamellar niosomal suspension. Niosomes derived from proniosomes have the ability to enhance the bioavailability of either hydrophilic or lipophilic drugs [5]. A study was carried out in which the vinpocetine proniosomes were prepared to analyze the effect of proniosomes on the bioavailability of poorly soluble drugs. The study concluded that proniosomes could improve the gastrointestinal absorption of vinpocetine and can provide an effective mean of delivering poorly water-soluble drugs through the oral route [6]. Proniosomal system also exhibited an improvement in the oral bioavailability of isradipine [7].

Niosomes are considered to be among the most suitable drug delivery systems discovered. It was first used within the cosmetic delivery until the self-composition behaviour of the non-ionic surfactant grabbed the attention of the pharmaceutical industry community.

Most of the studies that have addressed the behaviour of niosomes (the hydrated product of proniosomes) indicated that the pharmacokinetics of the drug has changed drastically after the inclusion into the vesicular carrier system. Drugs incorporated into the proniosomes have shown increased solubility, which in turn resulted in enhanced absorption of the drug [3].

The main components of proniosomes are a surfactant, membrane stabilizer, and carrier [8]. Carriers permit the flexibility in the ratio of surfactant and other components incorporated. they increase the surface area and enhance efficient loading e.g., sorbitol, mannitol, glucose, lactose [8,9].

Aceclofenac, a nonsteroidal anti-inflammatory drug (NSAID), has been indicated for various painful indications and proved to be as effective as other NSAIDs with lower indications of gastrointestinal adverse effects and, thus, resulted in greater compliance with treatment [10]. However, due to its poor bioavailability, it has a limited presence in the market [11].

This research came as an attempt to prepare Aceclofenac proniosomes successfully, to study different characterizations that can indicate the compatibility between ingredients and ensure the physical phase transformation required for better drug solubility and, hence, absorption. However, the ideal situation would be transforming proniosomes into niosomes whenever required to facilitate the production of various dosage forms. This is why the proper method of proniosomes hydration and factors affecting the hydration method of niosomes were studied. Also, this study focuses on different carriers that can be used in such preparation, and comparison of different formulations that have different carriers has been included.

## 2. Materials and Methods

### 2.1. Materials

Aceclofenac (COS Grade) was received as a gift sample from MEDA PHARMA (Dubai, United Arab Emirates), Glucose (D) and Mannitol (Analar) was purchased from BDH (London, England), Maltodextrin was purchased from Himedia Laboratories (Mumbai, India), Cholesterol was from MP Biomedicals, LLC Span 60, Chloroform and Methanol were from Merck (Darmstadt, Germany) and provided from IIUM, Malaysia. Disodium hydrogen phosphate, potassium dihydrogen phosphate, and sodium chloride were procured from Fluka Analytical (Seelze, Germany). All chemicals used in the study were of analytical grade.

### 2.2. Methods

#### 2.2.1. Preparation of Proniosomes

To prepare proniosomes, using the slurry method, three different carriers, namely maltodextrin, glucose, and mannitol, were used [12]. The Aceclofenac was dissolved in a mixture (1:1) of chloroform and methanol, then span 60 and cholesterol were dissolved in the same mixture. The completely dissolved mixture was transferred to a 250 mL rounded flask containing the required amount of the carrier. Then, the mixture was dried completely at 40 °C at 80 rpm under vacuum (16 mm Hg) using a rotary flash evaporator (IKA, HB 10 Basic, Staufen, Germany).

In all the formulations, the composition of proniosomes preparation was dependent on the actual dose of aceclofenac in the market, which is 100 mg. So, the composition of each formula was 100 mg Aceclofenac, 500 mg span 60, 250 mg cholesterol; the carrier amount was kept at 66% *w*/*w*, which is 1500 mg per dose while the solvent mixture selected was methanol and chloroform in the ratio of 1:1 *v*/*v*.

#### 2.2.2. Characterization of Proniosomes

##### Fourier Transform Infrared (FTIR) Analysis

To study the interaction between the different ingredients used in the formulation of proniosomes a PerkinElmer UTAR and Two FT-IR spectrophotometers was employed [13]. The spectra was studied and the peaks were analyzed for the interaction compatibility between the ingredients used and ensured the absence of any interactions that change the drug molecule. Following this procedure, the analysis of the pure drug, individual carrier, physical mixture of all ingredients, and the different formulas was done. The samples were placed onto the surface of the Zinc selenide crystal and forced gauge between 80–90 was applied with a pressure arm to ensure complete contact between samples and the crystal to get the most accurate results. Finally, samples scanning by using PerkinElmer Spectrum software (Model 1600, Massachusetts, US) were carefully conducted.

##### Differential Scanning Calorimetry (DSC) Analysis

Differential scanning calorimetry analysis was performed for pure aceclofenac, ingredients used, and prepared proniosomes to record any melting point changes between the pure drug and the formulations prepared. During this procedure, a measured amount of 6–10 mg of samples, namely aceclofenac and optimized proniosomes formulations, were studied. The samples were placed in aluminum crucibles and a reference was also used as a blank aluminum crucible, with a constant flow of nitrogen at 20 mL/min. The scanning range was between 80–220 °C at a heating rate of 10 °C/min and nitrogen was used as a purge gas by the use of Mettler Toledo (Ohio, USA) differential scanning calorimeter, model DSC 3 [14].

##### Powder X-ray Diffractometry (XRD) Analysis

Using X-ray diffractometry (XRD), the crystallinity of the pure drug optimized proniosome powder was measured with different carrier formulations using an X-ray diffractometer (X’ Pert PRO PANalytical, West Borough, MA, USA) using CuKα radiation, nickel-filtered graphite monochromator at 45 kV voltage, and 40 mA current with X’celerator detector for the purpose of measurement. The samples were analyzed at small-angle X-ray scattering (SAXS) runs with the samples at 1° (2θ) min−1 from 3° to 60° (2θ). The crystallinity of the formulation was recorded, with peaks appearing after the analysis with pure aceclofenac and formulations.

##### Scanning Electron Microscopy (SEM)

The Scanning electron microscopy (SEM) is a microscope that uses electron beams to produce an accurate image of the sample, through focusing the beam of electrons to interact with the atoms producing various signals that are translated into information regarding the topography and the composition of the sample. The topography (surface morphology), as well as the proniosome’s shape, was studied using the SEM. Proniosome powders (FN1, FN2, and FN3) were examined separately by SEM. Each sample was sprinkled over an aluminum stub using double-sided adhesive carbon tape, then samples were stored under a vacuum until total removal of air in ‘Leica Em SCD005′ sputter coater. The samples were sputter-coated with gold for 60 s to get a thickness of 14 nm. After the coating, the surface morphology (roundness, smoothness, and formation of aggregates) of proniosomes was scanned by Carl Zeiss AG-EVO^®^ 50 Series under a magnification power that ranged from 50 x to 4k x [15].

##### Proniosomes Flowability

The flowability of the prepared proniosomes was found through the bulk density and the tapped density of the proniosomal powder and then further related to Carr’s Index and Hausner Ratio (HR) [16] Carr’s index. The following index was used to compute tapped and bulk density:

Carr’s index = (Tapped Density−Bulk Density/Tapped Density) × 100

Hausner ratio: This was calculated from tapped and bulk density using the following expression:

Hausner ratio = Tapped Density/Bulk Density.

#### 2.2.3. Hydration of Niosomes and Optimization of Hydration Conditions by Using %EE

The proniosomes were prepared primarily to obtain niosomes with high entrapment efficiency upon hydration. During this process, two factors were studied, the time and volume of the hydrating medium in order to get the best conditions that should be applied for the hydration process. For further studies, the niosomes with relatively higher entrapment efficiency were selected.

Following Solanki et al. [17] a 3-level factorial design was used [18] in which the volume of the hydrating medium and time for hydration were the independent factors, while entrapment efficiency was the response factor. The procedure for hydration of proniosomes was similar to what song and Tian have mentioned with little modifications [6]. The proniosomes were hydrated using different volumes of warm distilled water (80 °C) [2]. via hand shaking technique and niosomes kept for different timings according to the experimental design mentioned in Table 1. The entrapment efficiency was evaluated using indirect method. Entrapment efficiency was determined by using the centrifugation methods, in which 1 mL of the prepared niosomal suspension was placed in the eppendorf for centrifugation by using MESM EBA200 Table Top Centrifuge at 1400 rpm for 1 h. All supernatant was separated into 10 mL volumetric flasks, then the sediment was resuspended with distilled water twice to ensure that all the unentrapped drug removed, all the supernatant was added together in the 10 mL volumetric flask, and distilled water was added up to the mark (dilution factor = 10). Peak area was measured using HPLC at 276 wavelength, and all formulas were done in triplicate [18].

$$\begin{array}{l} \mathrm{Entrapment}\;\mathrm{efficiency}\;\left(\mathrm{EE}\%\right)\\ =\frac{\left(\mathrm{Total}\;\mathrm{amount}\;\mathrm{of}\;\mathrm{the}\;\mathrm{drug}\;\mathrm{in}\;\mathrm{the}\;\mathrm{dosage}\;\mathrm{form}\;\mathrm{unit}-\mathrm{Amount}\;\mathrm{of}\;\mathrm{the}\;\mathrm{unentrapped}\;\mathrm{drug}\right)\times 100}{\mathrm{Total}\;\mathrm{amount}\;\mathrm{of}\;\mathrm{the}\;\mathrm{drug}\;\mathrm{in}\;\mathrm{the}\;\mathrm{dosage}\;\mathrm{form}\;\mathrm{unit}} \end{array}$$

Entrapment efficiency was determined, and results were analyzed using Microsoft Excel 2016 and factorial ANOVA using IBM SPSS^®^ statistics version 20, Chicago, IL, USA.

Amount of drug entrapment was measured using HPLC analysis [19,20]. HPLC method has been done according to Paul [21]. The HPLC used was Shimadzu Prominence model CTO–10AS VP with the column of Hypersil Gold, Dim (mm) 250 × 4.6, with the following chromatographic conditions, mobile phase: Phosphate buffer, (pH 5.0): Acetonitrile with a ratio: 60:40 (*v*/*v* A and B), wavelength of detection: 275 nm, flow rate 1.0 mL/min, and column oven temperature: 20 °C.

#### 2.2.4. Characterization of Niosomes Derived from Proniosomes

Formulated proniosomes were hydrated with distilled water to get the niosomes and it was further characterized by the following studies.

##### Vesicle Size, PDI, and Zeta Potential Measurements

The vesicle size was measured for the different niosome formulations using Zetasizer (NANO-S Malvern Instrument, Worcestershire, UK). Then, 0.2 mL of niosomes were diluted in ultrapure water (1.9 mL) and placed it in disposable sizing cuvette for measurement and samples run at the temperature of 25 °C at 173° scattering angle. The average diameter and the polydispersity index (PDI) were determined in triplicate.

Zeta potential for samples was measured using (NANO-Z Malvern Instrument, UK), the same dilution of niosomal suspensions was used, and measurements were conducted on the same temperature by the use of folded capillary cell for surface charge measurement [22].

##### Drug Content

For drug content measurement, 1 mL samples were obtained from the suspension and then placed in 100 mL volumetric flask; the volume was made up by using propranolol. Then, the samples were filtered through a 0.45 µm membrane and analyzed using HPLC, following the same method mentioned above [11]. Results were analyzed and computed by using Microsoft Excel 2016.

##### Optical Microscopy

Optical microscope Leica DM750 (Wetzlar, Germany) with an attached camera was used for image capturing, and niosomes were examined for their shape and size [23].

##### Statistical Analysis

All results were expressed as mean values ± SD (n = 3). For comparisons, one-way ANOVA test was employed to determine statistically significant differences at a significance level of *p* < 0.05. These tests were performed using IBM SPSS^®^ statistics version 20.

## 3. Results and Discussion

### 3.1. Proniosomes Characterization

#### 3.1.1. Fourier Transform Infrared (FTIR) Analysis

**Figure 1:**
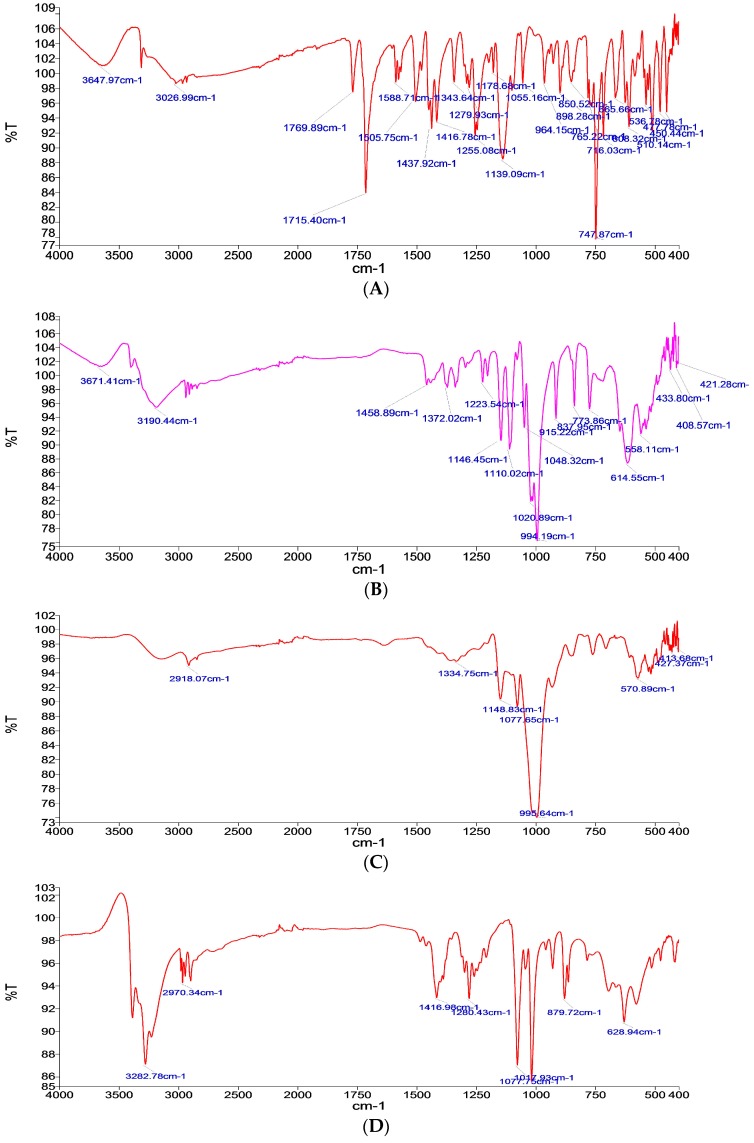

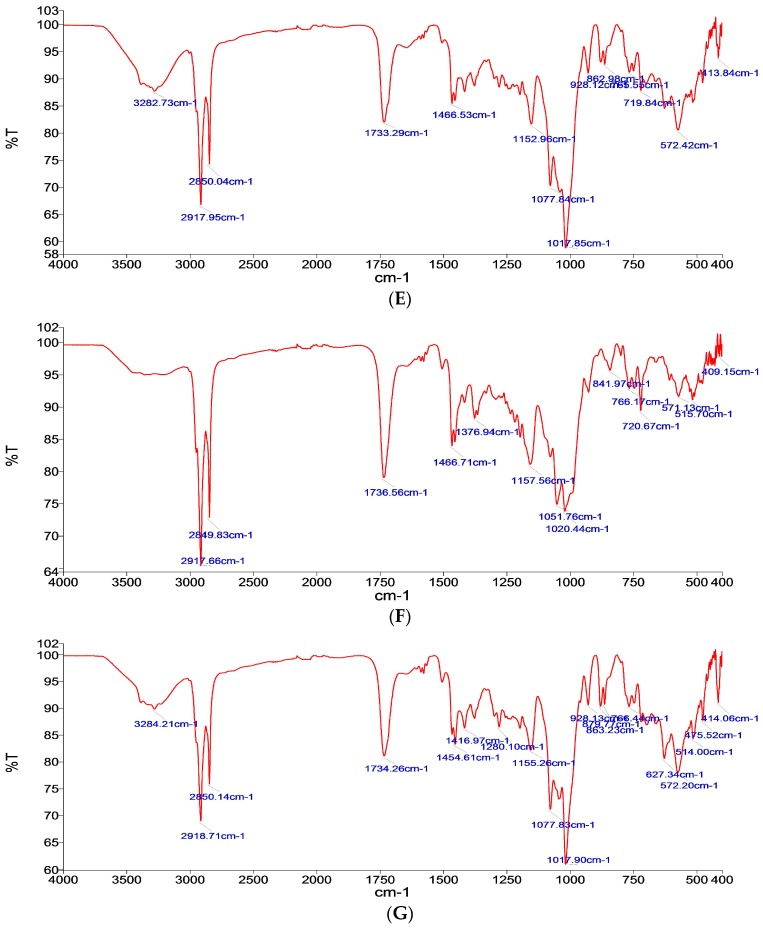
Fourier Transform Infrared FTIR Spectra of (**A**) pure drug, (**B**) glucose carrier, (**C**) maltodextrin carrier, (**D**) mannitol carrier, (**E**) formulation with glucose as a carrier (FN1), (**F**) formula with maltodextrin as a carrier (FN2), and (**G**) formula with mannitol as a carrier (FN3).

The FTIR results analyzed the IR spectrum of pure aceclofenac as in Figure 1A, which shows many characteristic bands such as at 787.87 cm^−1^, 1139.09 cm^−1^, 1416.78 cm^−1^, 1437.92 cm^−1^, and 1715.40 cm^−1^; these results are in-line with Girma [18].

Meanwhile, the IR spectrum for the used carriers, as shown in Figure 1B–D, are as the following: Glucose characteristic bands are at 994.19 cm^−1^, 614.55 cm^−1^, and 1020.89 cm^−1^, [24] maltodextrin characteristic bands are at 995.64 cm^−1^ and 570.89 cm^−1^, which characterizes the anhydroglucose ring stretching vibrations [25], and mannitol characteristic bands are at 1017.93 cm^−1^, 1077.75 cm^−1^, and 3282.78 cm^−1^ [26].

The absence of any significant change in the IR spectrum pattern in the formulations containing the drug and carrier, as in Figure 1E–G, confirms the absence of any interaction between the drug and carrier used, hence it can be concluded that no interaction was determined between glucose, mannitol, or maltodextrin and all the other components used, respectively.

#### 3.1.2. Differential Scanning Calorimetry (DSC) Analysis

**Figure 2:**
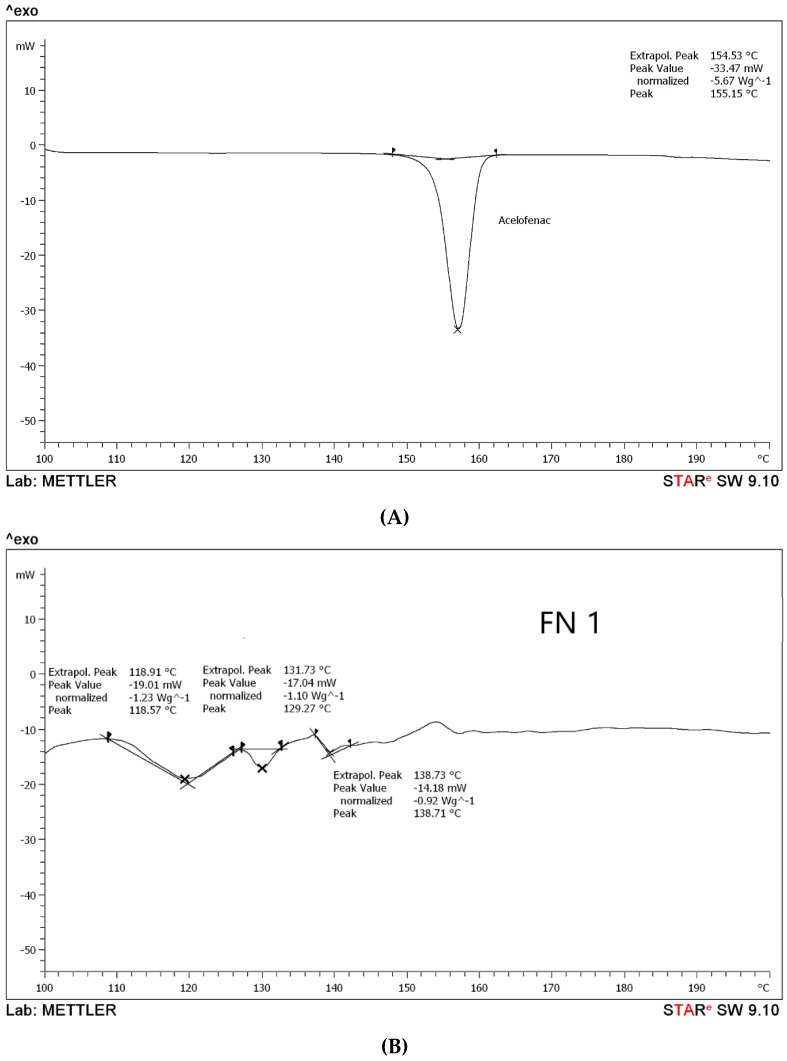

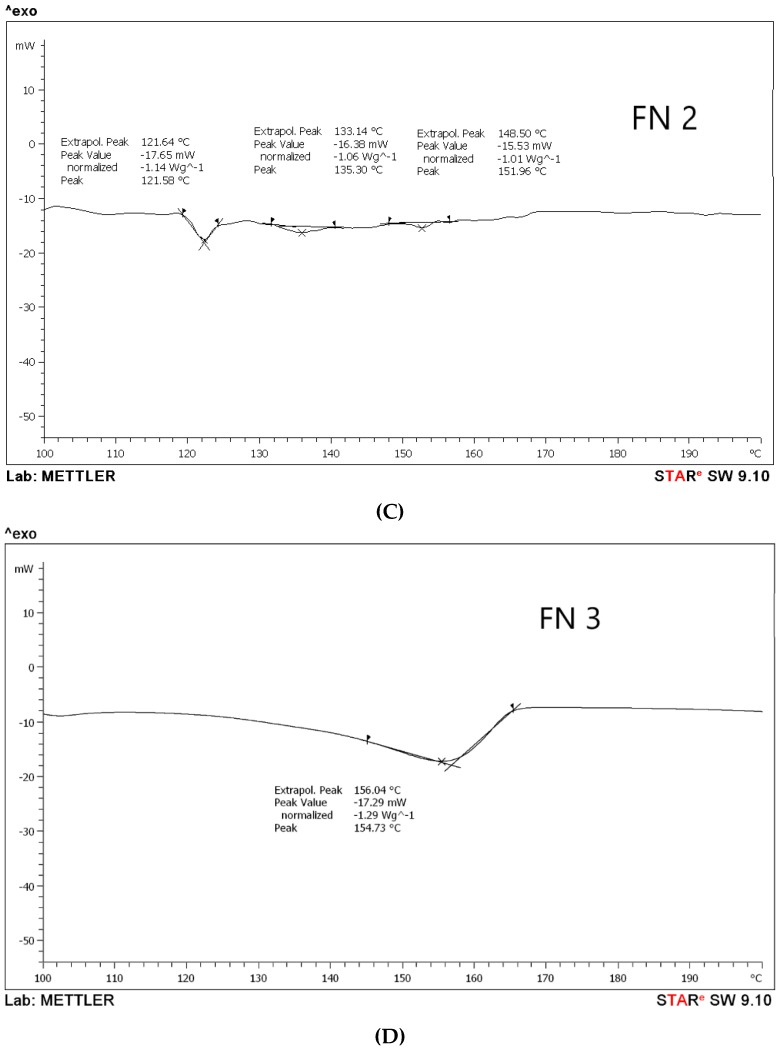
Differential scanning calorimetry (DSC) thermograms of (**A**) pure drug, (**B**) formula with glucose as a carrier (FN1), (**C**) formula with maltodextrin as a carrier (FN2), and (**D**) formula with mannitol as a carrier (FN3).

According to the DSC results, it has been observed that aceclofenac shows an endothermic sharp peak at 155.15 °C with a peak value −33.47 Mw, as in Figure 2A, which is the melting point of aceclofenac. Further, the melting points for different proniosomal preparations were recorded as the following, according to Figure 2B–D. FN1 recorded at 118.91 °C, 131.73 °C, and 138.73 °C with peaks varied between −14.18 Mw to −19.01 Mw), while FN2 endothermic peaks are 121.64 °C, 133.14 °C, and 148.50 °C with peak values ranging between −15.53 Mw to –17.65 Mw, and FN3 has been recorded at 156.04 °C with a peak value of –17.29 Mw. According to the peak values, it is clear that the sharp peak is absent in all proniosomal formulations. Although, the endothermic peaks of the carriers are for glucose at 155.8 °C [27], maltodextrin at 160 °C [15], and mannitol at 167.8 °C [28]. Consequently, decreasing the melting point in proniosomes with glucose, maltodextrin, and mannitol, as shown in Figure 2, revealed that a physical change occurred in aceclofenac proniosomal formulations. A research study carried out by Veerarddy and Bobbala [7] revealed that the absence of a sharp peak explains that the physical state of aceclofenac changes from crystalline to the amorphous phase, which proves the enhanced solubility and faster dissolution.

The dull peaks presented in Figure 2B–D for FN1, FN2, and FN3 could be attributed to the existence of a degree of crystallinity due to the presence of the carriers, different carriers used as glucose and maltodextrin, and also to the possibility that mannitol led to different dull endothermic peaks [14]. The study was only done on the formulated proniosomes as our concern is to observe the effect of different carriers and their effect on the formulation.

#### 3.1.3. Powder X-ray Diffractometry (XRD) Analysis

**Figure 3:**
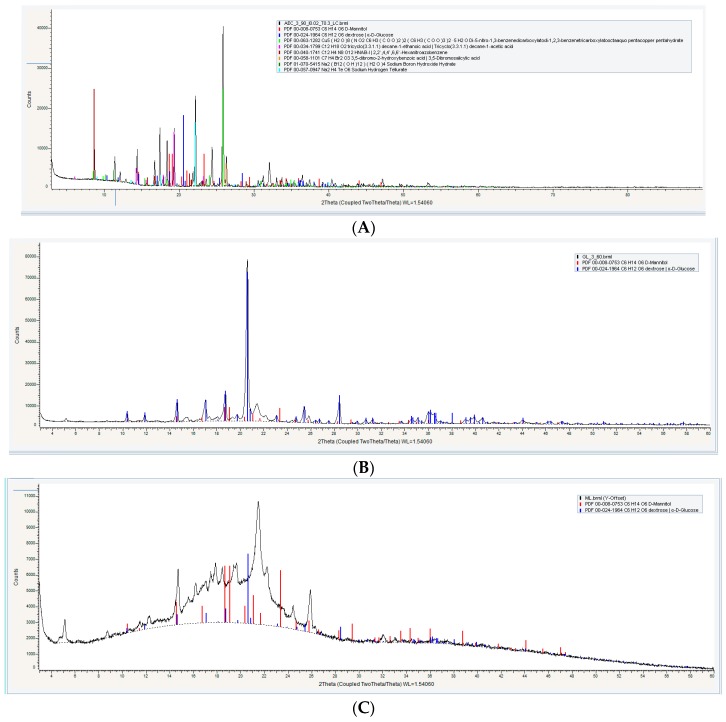

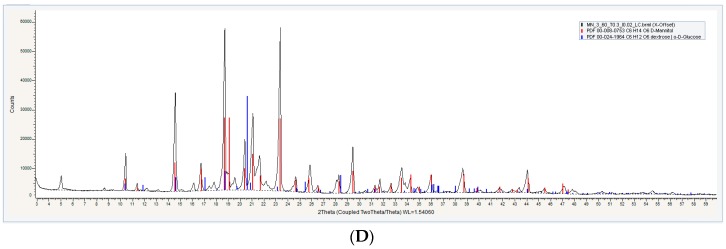
Powder X-ray Diffractometry XRD of (**A**) pure drug, (**B**) formula with glucose as a carrier (FN1), (**C**) formula with maltodextrin as a carrier (FN2), and (**D**) formula with mannitol as a carrier (FN3).

For properly assessing the degree of crystallinity of the proniosome constituents, powder X-ray diffractometry (XRD) analysis was applied. It has been observed that the aceclofenac pure drug reached high peaks at 27° and 22°, respectively, as in Figure 3A, and also shows some peaks from 10 θ to 20 θ, which correspond to crystal nature of the aceclofenac at (2 θ) [29].

According to different literature, carriers (glucose, maltodextrin, and mannitol) exhibited differences in their crystallinity; glucose is considered a crystalline substance that has a sharp peak at 18° (2 θ) [30], while maltodextrin exhibited no crystalline characteristic peaks; it has been observed by many authors that if the peaks are not sharp, then the crystalline nature does not exist [31] and mannitol is considered a highly crystalline substance with XRD high peaks at 9.6°, 16.5°, 18.0°, and 25.7° at (2 θ) [32].

Proniosomal formulations exhibited differences in the XRD analysis, as shown in Figure 3; FN1 and FN3 show a high peak, however these peaks are not the significant diffraction peaks of aceclofenac, while maltodextrin gave a board curve. These findings support the hypothesis that proniosome formulations have the ability to decrease the degree of crystallinity of the pure drug and enhance its amorphous nature [33].

The presence of other sharp peaks in FN1 and FN3, as in Figure 3B,D, is due to the presence of glucose and mannitol as a carrier, which has a crystalline state. From the crystalline behavior it is inferred that there is entrapped in the bilayer membrane [6].

Results suggested that the degree of crystallinity significantly affected the solubility of the proniosomes upon hydration. As shown in Figure 3C, FN2 exhibited an amorphous state much more than FN1 and FN3 [34]. This means that maltodextrin is more soluble than other carriers.

Solubility of the carriers used may affect the dissolution and absorption of the drug after oral administration. In vitro and in vivo studies should be carried out to confirm the effect of the carriers’ solubility on the drug absorption and, hence, bioavailability.

#### 3.1.4. Scanning Electron Microscopy (SEM)

**Figure 4:**
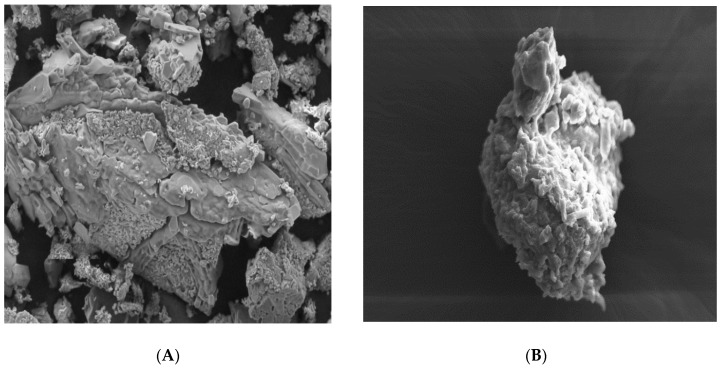

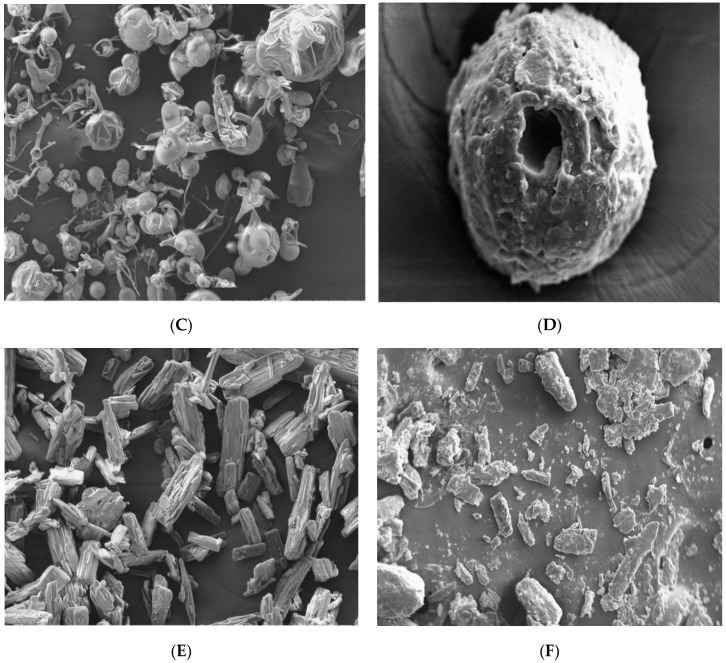
Scanning Electron Microscopy SEM Images of (**A**) pure glucose carrier, Magnification (Mag) = 250 X, (**B**) glucose ptoniosomes FN1, Mag = 500 X, and (**C**) Pure maltodextrin, Mag = 250 X, (**D**) maltodextrin proniosomes, FN2, Mag = 445 X, (**E**) pure mannitol carrier, Mag = 250 X, (**F**) mannitol proniosomes, FN3, Mag = 100 X.

The topography (surface morphology) of aceclofenac, different carriers (glucose, maltodextrin, and mannitol) and proniosomes formulations (FN1, FN2, and FN3) has been examined and revealed that all carriers have been coated properly and adequately according to Figure 4B,D,E.

Pure aceclofenac is normally characterized by its crystal shape and smooth surface [35,36], while glucose appeared with irregular shape, as in Figure 4B, maltodextrin had a spherical shape and was characterized by holes spreading over its surface [37] while mannitol had a crystalline, cylindrical shape, [38] but all carriers had a smooth surface, as shown in Figure 4A,C,E.

In this study, and according to the SEM results, proniosomes with glucose, maltodextrin, and mannitol as carriers were obtained under the SEM with proper coating as in Figure 4B,D,E. A significant difference between the carriers before coating and after coating were clearly observed.

All particles of the proniosomal formulations were completely coated with the surfactant as in Figure 4B,D,F. All images support the presence of the surfactant (span 60) layer with its rough surface over the particles that ensures a high entrapment efficiency of the niosomes derived from proniosomes after hydration. However, a thick layer of coating with the presence of granular surface suggests that proper hydration and agitation should be done for the proniosomes for ensuring proper niosomes formation [39]. According to Abd Elbary (2008), the degree of thickness does not negatively affect the entrapment efficiency [40].

#### 3.1.5. Powder Flowability

Flowability results indicate that all formulations were smooth with good flowability as in Table 2, and non-significant differences were found between the flowability of glucose, maltodextrin, and mannitol proniosomes as presented in Table 2. These results were supported by other researchers who have studied the flowability of proniosomes [41,42]. Also, using the slurry method for the preparation of proniosomes confirmed the extraction of a good flowable proniosome powder [8].

A free flowable powder is considered a desirable outcome for further processing as a tablet or capsules, as well as for packaging and pouring of the powder before its use [43].

### 3.2. Effect of Volume of Hydration and Time of Hydration

Our aim was to set entrapment efficiency as a dependent factor for the reason that proniosomes are mainly judged on the basis of the entrapment efficiency. That is why one carrier has been chosen randomly, which is maltodextrin, in order to optimize the hydration method of the proniosomes. Hydration of proniosomes to niosomes is a significantly important factor that can possibly affect the entrapment efficiency of the prepared niosomes. For this purpose, the volume of hydration and time of hydration were studied to optimize the proper conditions of niosomes’ hydration.

According to the three-level experimental design in Table 1, entrapment efficiencies of nine niosomal formulations (FM1–FM9) were compared. The results revealed that the volume of the hydrating medium had a significant effect (*p* < 0.05) on EE%; the smaller the hydrating volume, the better EE%, as shown in Table 3. These findings are in line with research that was done in 2010 by Ruckman [44], which relates the high entrapment efficiency with a low volume of hydration and concluded that leakage of the drug from niosomes increases with increase in the volume of hydration. FM1 exhibited the highest EE%, which is 84%, in comparison to FM9 with 73% only.

**Figure 5:**
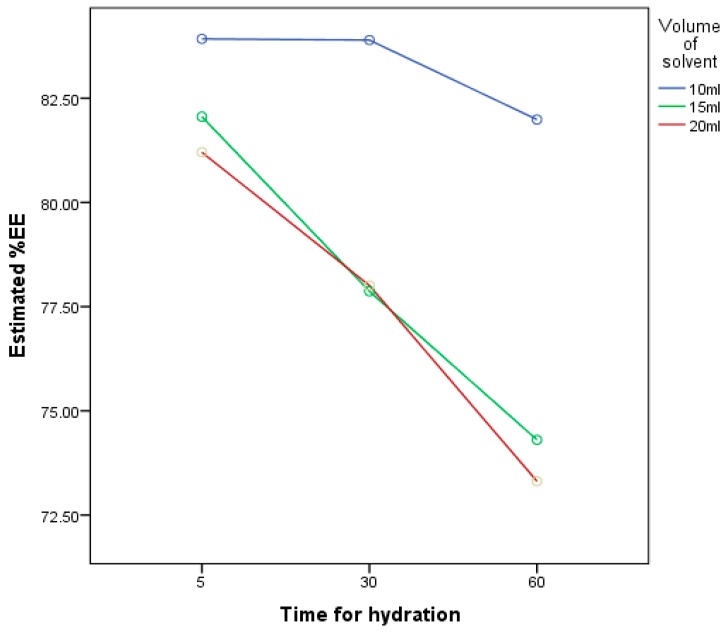
A graph representing the relationship between the results of the three-level factorial design that has been studied for optimization of hydration conditions; the factors were: The volume of the hydrating medium and the time for hydration, and the response is their effect on the % of the entrapment efficiency.

The present study confirms that time of hydration and volume of hydration has a significantly negative effect (*p* < 0.05) on %EE according to Figure 5. With an increase in time and volume of hydration, %EE decreases. At 10 mL hydration volume, the highest %EE was observed with no significant difference (*p* > 0.05) between 5 min and 30 min of hydration, although other researchers related the time of hydration with the entrapment efficiency by claiming that the more the niosomes hydrated the more the drug entrapped [44].

The time of niosomes hydration has been differently mentioned in the early scholarly writings. Most of the hydrated niosomes were kept for 2 min [45], while some researchers prefer to hydrate the niosomes for 30 min [46] and other researches continued the hydration for 60 min [41]. Most of the above-mentioned studies revealed that the time of hydration affects the shape and size of the vesicle without any relation to the entrapment efficiency.

The findings of this research supported the idea of the study that because proniosomes should be hydrated before administration, less waiting time will increase the applicability of the product and will enhance patient compliance.

### 3.3. Characterization of Niosomes Derived from Proniosomes

#### 3.3.1. Entrapment Efficiency

The results revealed a high percentage of aceclofenac that was entrapped in the niosomes formed; mostly this is consequent to the use of span 60, which was chosen among all the other span types due to its long alkyl chain that result in higher entrapment efficiency if compared with the other types of span [47]. Regarding the type of carrier used, non-significant results were recorded between FN1, FN2, and FN3, as illustrated in Table 4. No differences were observed between the different formulas that have different carriers: Glucose, maltodextrin, and mannitol. All carriers were able to give a high entrapment efficiency with non-significant differences.

The explanation beyond this finding could be due to the cholesterol used in the formula. As cholesterol is considered as an amphiphilic molecule, it orients its OH group towards aqueous phase and aliphatic chain towards surfactant’s hydrocarbon chain. Therefore, the multilamellar vesicles could be formed with proper rigidity; the large volume of the aqueous hydrating medium used could decrease the rigidity of the formed vesicles. Weak vesicles will have fewer tendencies to encapsulate the drug and low entrapment efficiency would appear [48].

#### 3.3.2. Vesicle size, PDI, and Zeta Potential Measurements

Vesicle size is a very critical attribute of lipidic nanocarriers, which affects stability, entrapment efficiency, bioavailability, bio-distribution, and cellular uptake. Techniques used in the preparation of such systems play a vital role in controlling vesicle size and distribution [48].

Membrane additives will significantly increase the stability of the produced niosomes. Each component used in the system such as the surfactant, the membrane stabilizers, etc. will greatly affect the morphology of the vesicles and the release of the drug from the vesicles.

According to our results, niosomes derived from proniosomes were analyzed with an average diameter of 4–6 µm, in Table 4, which is considered a large vesicle in comparison to other niosomes; the formation of a large vesicles came as a result of well hydration coupled with proper agitation through hand shaking technique, which is considered applicable and simple. Increased agitation force is the prime reason for the reduction of the vesicles size as in the sonication technique [49], although sonication was used in a number of previously done research in the current area of interest [49].

The polydispersity index (currently known as dispersity index) is a measure of the distribution of the particle size of a certain sample (heterogenicity of the size).

Our results revealed PDI values ranging 0.58–1, as in Table 4, which indicates variation in the particle size distribution in all formulations [50]. Glucose shows the lowest PDI, which means the highest homogeneity in the niosomal dispersion, while mannitol explores the highest value of the PDI, which is 1.0 and indicates a heterogenous niosomal system.

Heterogenicity of the vesicle size in all niosomal formulations is due to the large size of the niosomes prepared, which is the main reason for higher differences in the size distribution in comparison with small vesicles. Meanwhile, vesicles with larger size have a higher tendency toward the drug entrapment.

The zeta potential results for all the niosomes formed (FN1, FN2, and FN3) were high and had non-significant differences, as in Table 4. The absolute value of the zeta potential is an indicator of the niosomes’ stability; the larger the zeta potential absolute value, the larger the amounts of surface charge, which means increased repulsive interactions that led to more stable particles with a more uniform size distribution [5,51]. Also, using cholesterol as a membrane stabilizer increases the rigidity of the vesicular bilayer and results in decreasing the release of the drug from the niosomal system, which enhances niosomes’ stability.

#### 3.3.3. Drug Content

The drug content estimation within the niosomes is an essential step during the characterization process; drug content ensures that every dose contains the amount of active ingredient intended with little variation among other doses within a batch.

The niosomes prepared in this study revealed 96–100% drug content. These findings were in all formulations that were prepared with different carriers. As shown in Table 4, non-significant differences were observed between glucose, maltodextrin, and mannitol proniosomes. These findings indicate the appropriateness of the method of preparation and support the hypothesis of the research, which introduces different carriers in the proniosomes’ preparation without any significant differences [10].

#### 3.3.4. Optical Microscope

**Figure 6:**
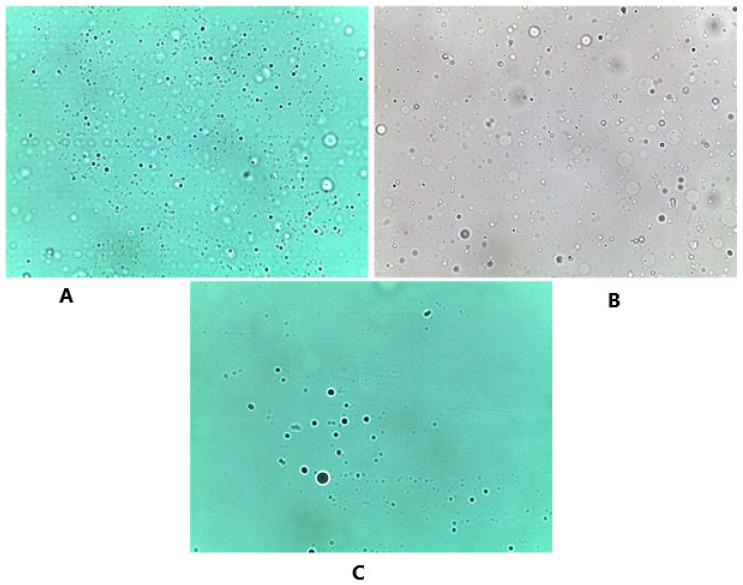
Optical microscope images that show the spherical shape of the formed niosomes after hydration, and the distribution of these niosomes. The three different carriers were examined under the microscope accordingly. (**A**) Glucose niosomes (FN1) Magnificatin (Mag) = 20 X, (**B**) maltodextrin niosomes (FN2) Magnification (Mag) = 20 X, (**C**) mannitol niosomes (FN3) Magnification (Mag) = 20 X.

The prepared niosomes appeared in Figure 6 as a large multilamellar vesicle with a spherical shape. Similar to what has been mentioned by Sivaprasad et al., [8] no aggregation or agglomeration was observed under the microscope and smooth appearance was indicated. These results indicate the preparation of well-hydrated niosomes and similar findings were also observed in the preparation of indomethacin proniosomes [5].

## 4. Conclusions

Aceclofenac proniosomes were prepared successfully by using different carriers such as glucose, mannitol, and maltodextrin. Physicochemical characterization concluded the possibility of preparing proniosomes by the use of different carriers, although maltodextrin exhibited the highest solubility among other carriers that may affect the bioavailability of the prepared formula. However, niosomal characterization concluded non-significant differences in term of entrapment efficiency, vesicle size, and content uniformity. Providing different alternatives for such formulation gives a good opportunity for the market to offer different products not only for aceclofenac, but also for any other drug with low bioavailability. Proniosomes can be prepared easily by the slurry method and niosomes derived from proniosomes prepared exhibited a niosomal suspension with good stability, high entrapment efficiency (84% ± 1), and (98% ± 4) drug content. Proniosomes can be hydrated with a minimum amount of water, which is acceptable for adult administration, and only 5 min wait is required for the patient to take their medication after hydration. The optimized aceclofenac proniosomes in this investigation need to be further explored for bioavailability and stability studies.

## Figures and Tables

**Table 1:** Three-level factorial design for optimization of hydration conditions.

Factor	Level			Response
	−1	0	1	
The volume of hydration (mL)	10	15	20	Entrapment Efficiency
Time for hydration (min)	5	30	60	

**Table 2:** Representing the micromeritic properties of various proniosomal powder formulations, which includes the bulk and tapped density of all formulations and their corresponding flowability according to the Carr’s Index and Hausner’s ratio.

Formulation Code	Bulk Density (g/mL)	Tapped Density (g/mL)	Carr’s Index	Hausner’s Ratio	Type of Powder
FN 1	0.36 ± 0.01	0.43 ± 0.01	16.6 ± 0	1.2 ± 0	Good Flowable
FN 2	0.22 ± 0.04	0.28 ± 0.05	19.4 ± 1.3	1.24 ± 0.02	Fairly Flowable
FN 3	0.27 ± 0.02	0.38 ± 0.01	21.7 ± 2.5	1.31 ± 0.04	Fairly Flowable

**Table 3:** The following table illustrates the results of the three-level factorial design that has been studied for optimization of hydration conditions; the factors were: The volume of the hydrating medium and the time for hydration, and the response is their effect on the % of the entrapment efficiency.

Std Order	Formulas	The Volume of Hydrating Medium	Time for Hydration	%Entrapment Efficiency
1	FM 1	10 mL	5 min	84 ± 0.66
2	FM 2	10 mL	30 min	84 ± 0.75
3	FM 3	10 mL	60 min	82 ± 0.28
4	FM 4	15 mL	5 min	82 ± 0.29
5	FM 5	15 mL	30 min	78 ± 0.66
6	FM 6	15 mL	60 min	74 ± 0.74
7	FM 7	20 mL	5 min	81 ± 0.94
8	FM 8	20 mL	30 min	78 ± 0.61
9	FM 9	20 mL	60 min	73 ± 0.31

**Table 4:** Illustration of different characterization of the niosomes derived from proniosomes, which includes the % of entrapment efficiency, size of the formed niosomes in nm, their Pdl and Zeta potential, and the % of the drug content in the niosomal suspensions formed.

Formulation Code	%EE ± Standard Deviation (SD)	Z-Average ± SD (d.nm)	Pdl	Zeta Potential ± SD (mV)	% of Drug Content ± SD
FN 1	82 ± 0.5	5240 ±128	0.58	−46.3 ± 5.96	101 ± 0.65
FN 2	84 ± 0.66	4669 ± 20	0.7	−45.2 ± 5.14	98 ± 4
FN 3	84 ± 0.34	6403 ±25	1	−48.5 ± 5.06	96 ± 8
